# CT gel dosimetry technique: Comparison of a planned and measured 3D stereotactic dose volume

**DOI:** 10.1120/jacmp.v3i2.2575

**Published:** 2002-03-01

**Authors:** C. Audet, M. Hilts, A. Jirasek, C. Duzenli

**Affiliations:** ^1^ British Columbia Cancer Agency British Columbia Canada V5Z 4E6; ^2^ Palo Alto Medical Foundation Palo Alto California 94305; ^3^ Department of Physics and Astronomy University of British Columbia Vancouver Canada V5Z 4E6

**Keywords:** Polymer gel, Stereotactic radiosurgery, 3D dosimetry, X‐ray CT

## Abstract

This study presents a 3D dose mapping of complex dose distributions using an x‐ray computed tomography (CT) polymer gel dosimetry technique. Two polyacrylamide gels (PAGs) of identical composition were irradiated with the same four arc stereotactic treatment to maximum doses of 15 Gy (PAG1) and 8 Gy (PAG2). The PAGs were CT imaged using a previously defined protocol that involves image averaging and background subtraction to improve image quality. For comparison with the planned isodose distribution, the PAG images were converted to relative dose maps using a CT number‐dose calibration curve or simple division. The PAG images were then co‐registered with the planning CT images in the BrainLab® treatment planning software which automatically provides reconstructed sagittal and coronal images for 3D evaluation of measured and planned dose. The hypo‐intense high dose region in both sets of gel images agreed with the planned 80% isodose contour and was shifted by up to 1.5 and 3.0 mm in the axial and reconstructed planes, respectively. This demonstrates the ability of the CT gel technique to accurately localize the high dose region produced by the stereotactic treatment. The resulting agreement of the measured relative dose volume for PAG1 was within 3.0 mm for the 50% and 80% isodose surfaces. However, the dose contrast was too low in PAG2 to allow for accurate definition of measured relative dose surfaces. Thus, a PAG should be irradiated to higher doses if quantitative relative dose information is required. Unfortunately, this implies use of an additional PAG and its CT number dose response since doses greater than 8–10 Gy fall outside the linear regions of the response.

PACS number(s): 87.53.–j, 87.57.–s, 87.59.Fm

## I. INTRODUCTION

A current trend in radiation therapy dose delivery is towards highly localized, conformal techniques such as intensity modulated radiotherapy (IMRT) and stereotactic radiosurgery (SRS). These techniques yield complex three dimensional (3D) dose distributions and require dose verification in 3D. Furthermore, due to the high dose gradients produced by such treatments, stringent requirements are placed on the spatial resolution of a dosimeter used to verify these treatments. Polymer gel dosimetry attempts to meet the requirements of 3D radiation dose verification.

The effects of radiation on polymer systems have been documented as early as the 1950s.^1^ ^,^ ^2^ However, applications of polymer gels to 3D radiotherapy dose verification did not occur until 1993 when Maryanski *et al*.^3^ proposed a polymer gel dosimeter imaged with magnetic resonance imaging (MRI). Since this initial investigation several formulations of polymer gel have been proposed for use in radiotherapy (i.e., BANG® and variations thereof (see http://www.connix.com/~mgsinc), VIPAR^4^). PAG consists of an acrylamide monomer and *N,N′* methylene‐bis‐acrylamide (bis) crosslinker infused in a gelatin matrix. Upon reaction with a suitable catalyst (i.e., radiation) the monomers react together to form a cross‐linked polymer network. This polymer network is spatially retained in the gelatin matrix and, furthermore, the amount of polymer is related to the dose delivered to the gel.

Although a highly promising candidate for filling the current void in 3D dose verification, polymer gel dosimetry has not yet gained widespread clinical acceptance. This is due, in part, to the methods available for extracting the dose information from an irradiated PAG. MR imaging has remained by far the most popular method for dose information extraction. The technique has met with a certain amount of success. Specifically, in the area of conformal radiotherapy, stereotactic radiotherapy and IMRT several workers have applied polymer gels in conjunction with MRI to obtain 3D dose maps of these treatments.^5^ ^–^ ^12^ It is nonetheless clear that some challenges exist with respect to the proper analysis of gels.^13^ ^–^ ^16^ Furthermore, the accessibility of MRI scanners to radiotherapy clinics is often problematic and the use of scanners expensive. Hence, other imaging modalities have been explored.

Optical computed tomography (optical CT or OCT) has recently been applied to polymer gels.^17^ ^,^ ^18^ The technique is promising, however; traditional polymer gel formulations (i.e., PAG) suffer from high light scattering properties and hence low signal to noise in OCT maps. Furthermore, the technique has been limited, to date, to phantoms with cylindrical symmetry. It remains to be determined if this limitation can be overcome. Currently the technique is in developmental stages and, as such, clinical implementation is not routine.

Most recently, x‐ray computed tomography (CT), a prevalent imaging modality in radiotherapy, has been proposed as a technique for extracting dose information from PAG^19^ In the paper, the feasibility of the technique, as well as a protocol for CT imaging, was outlined. Although the signal‐to‐noise ratio (SNR) observed in the CT images was relatively low, the imaging protocol maximized the useful dose information obtainable from the images. For the purposes of the feasibility study and development of the imaging protocol, 2D dose maps were analyzed from a collection of calibration and treatment gels. The potential to extend this work to 3D dose mapping is clear. This paper outlines the application of the polymer gel x‐ray CT technique to 3D dose mapping of a four arc SRS treatment.

## II. MATERIALS AND METHODS

### A. PAG Preparation

A detailed account of the gel preparation technique employed by this group is given elsewhere.^20^ Briefly, since oxygen is a known inhibitor of polymerization in PAGs, the gels used for irradiation were manufactured in an in‐house built glove box purged with purified nitrogen gas (Praxair). All gels were composed of 3% (by weight) acrylamide monomer, 3% *N,N′* methylene‐bis‐acrylamide (bis) crosslinker (both of electrophoresis grade, Sigma Chemical Co, St. Louis, MO), 5% gelatin (∼300 bloom, Sigma Chemical Co.) and 89% purified water. Both gels used for the SRS irradiations (PAG1 and PAG2) consisted of 800 mL of gel contained in 1 L spherical glass flasks. A third gel, identical in composition to PAG1 and PAG2 but used for background subtraction purposes (see Sec. IIC), was manufactured under atmospheric conditions to ensure that it would not polymerize during CT imaging.

### B. Treatment planning and PAG irradiation

**Figure 1:**
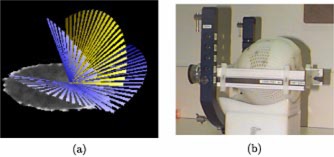
(Color) (a) The four arc SRS treatment used to irradiate both PAG1 and PAG2. (b) A PAG immobilized in the BrainLab® SRT mask and frame system.

A set of CT images was obtained of a water (see Ref. 21 for a note on the water equivalence of PAG) filled 1 L glass flask (identical to the PAG flasks) immobilized in the BrainLab® mask/frame system and with localizer box attached. These images were then imported into the BrainLab® treatment planning software and used as the planning CT image set for the PAG irradiations. A mono‐isocentric stereotactic treatment plan was designed that consisted of four arcs of circular, 30 mm diameter fields [see Fig. 1(a)]. This dose distribution was chosen as it provides a relatively simple distribution which is often used in our department.

The two active PAGs (PAG1 and PAG2) were each irradiated with the four arc SRS treatment ∼6h post manufacture. The treatment set up for the PAGs was identical to that used for imaging the water filled flask and is shown in Fig. 1(b). The irradiations for both PAGs were performed using the 6 MV beam (CL2100, Varian Associates, Palo Alto, CA). Each PAG was irradiated to a different maximum dose at isocentre: 15 Gy (PAG1) and 8 Gy (PAG2). One day post irradiation the PAGs were exposed to the atmosphere, at which time polymerization is thought to have terminated.^22^ The oxygen in the atmosphere neutralizes the PAGs and prevents CT x‐rays from potentially inducing polymerization.

### C. CT imaging

This group employed a conservative one week wait between exposing the irradiated PAGs to oxygen and CT imaging. This wait was more than enough to ensure oxygen had permeated through the entire gel volume^23^ and hence prevent further polymerization during CT imaging. All CT scanning was performed at room temperature (23 °C) using a single GE HiSpeed CT/*i* diagnostic CT scanner (Performix MX200, specific heat capacity 6.3 MHU). All images were obtained when the scanner was fully warmed up in order to reduce uncertainty in the CT number to dose response.^19^

**Figure 2:**
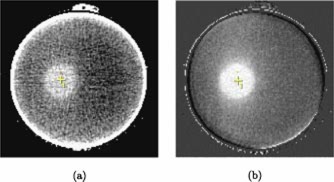
(Color) Axial images at isocenter illustrating the difference between the “co‐registration” and “dose analysis” CT image sets obtained for each gel. (a) The co‐registration image sets are characterized by high noise and artifacts caused by the fiducial markers (not shown) on the localizer box. (b) The final $\Delta N_{\text{CT}}$ images sets used for dose analysis cover solely the high dose region of the PAGs and have improved image quality due to omission of the localizer box, image averaging, and background subtraction.

Two sets of CT images were obtained for each of PAG1 and PAG2: (1) co‐registration and (2) a high quality CT set for the region of interest. Figure 2 shows an example image from each CT set. For both PAGs the co‐registration CT set consisted of images of the gel immobilized in the stereotactic mask/frame, including the fiducial marker localizer box system. This image set covered the entire gel volume. A standard set of CT imaging parameters were used (120 KV, 200 mAs, slice thickness and slice spacing of 3 mm) and one image was obtained for each scan position. These images of the gel contained in the localizer box system allowed for co‐registration of the gel images with the planning CT image set in the BrainLab® software. However, the gel image set was of too poor a quality for dosimetry purposes due to both high noise and beam hardening artifacts from the fiducial markers on the localizer box [see Fig. 2(a)].

A second set of higher quality images was obtained for the region of interest (the high dose region) in each gel. The same set of CT imaging parameters (above) were used for this set, but in order to reduce beam hardening artifacts, the fiducial marker box was removed during imaging. An mAs of 200 was chosen to maximize signal while maintaining the smaller filament mode which produced less image blurring. The quality of this high dose region image set was then further improved by using an image averaging and background subtraction protocol, described in detail in a previous paper.^19^ In brief, for each PAG, 16 images were obtained at each scan position throughout the high dose region and then averaged to reduce image noise. The number of images averaged, 16, was a compromise between improved image quality and imaging time. Furthermore, to reduce x‐ray tube heating and imaging time, only the high dose region was imaged in this manner. An unirradiated background gel geometrically identical to the active PAGs and positioned in the same set‐up was also imaged using the averaging protocol. These background images were then subtracted from the corresponding average PAG images to remove residual artifacts. The resulting change in CT number, $\Delta N_{\text{CT}}$, images of the high dose region in the PAGs have much improved image quality compared to the co‐registration CT images [see Fig. 2(b)] and provide useful dose information.

The final image quality and resolution were affected by the chosen slice thickness and spacing.^19^ A slice thickness of 3 mm was chosen to produce an acceptable signal to noise while minimizing the imaging time and tube heating associated with acquiring all of the images used for averaging. A slice spacing of 3 mm was chosen to maintain a reasonable overall imaging time (number of scan positions required to cover the high dose volume multiplied by the number of images taken per scan position for averaging purposes) at the expense of spatial resolution in nonaxial reconstructed planes. Faster more modern CTs with higher capacity x‐ray tubes could make decreasing slice spacing and thickness more feasible. Of note is the comparatively high axial spatial resolution of the CT images. Whereas CT images usually consist of 512×512 pixels, MR images usually consist of 256×256 pixels as 512×512 MR images require double the imaging time.

As the final imaging step, the two image sets for each PAG were combined to form a single set of CT images for PAG1 and likewise, a single set for PAG2. This was done by replacing the high dose region images in the first, co‐registration image set with the corresponding images from the second, high quality $\Delta N_{\text{CT}}$ image set [visible as central bands in Fig. 4(b)]. The result was a set of CT images for each PAG that had retained the fiducial markers required for co‐registration with the planning CT set and yet also included high quality images for extracting dose information.

**Figure 3:**
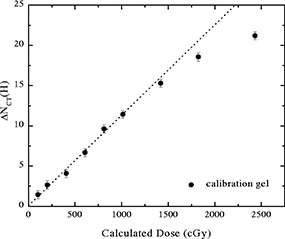
The $\Delta N_{\text{CT}}$‐dose response curve for a PAG calibration gel, reproduced here from Hilts et al.^19^ $\Delta N_{\text{CT}}$ increases approximately linearly with dose up to ∼10−12 Gy and then increases more slowly at higher doses.

**Figure 4:**
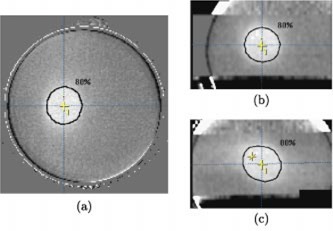
(Color) (a) Axial, (b) coronal, and (c) sagittal CT images of PAG1 (irradiated to a maximum dose of 15 Gy) at isocenter. The coronal and sagittal images were reconstructed from the set of axial gel images (3 mm slice spacing). The planned 80% isodose contour and a cross indicating the isocenter are marked in each image.

### D. Dose image derivation

The high quality, high dose region $\Delta N_{\text{CT}}$ images were converted to measured dose images for comparison with the planned high dose volume. The $\Delta N_{\text{CT}}$‐dose response (Fig. 3) used for the conversion was obtained using a cylindrical 1.5 l PAG irradiated with 2 cm diameter 6 MV photon beams. It is nonlinear with an approximate linear region up to about 10 Gy. As a result, relative dose images for the PAG2 (max. 8 Gy) gel were obtained without use of the calibration curve and simply by dividing the images by the maximum $\Delta N_{\text{CT}}$ (8 Gy). Since PAG1 was irradiated beyond the linear region of the response the relative dose images required use of the full calibration curve. To compare measured and planned relative dose information, the measured dose image pixel values were binned into relative dose ranges of <30%,30–50%,50–80%, and >80%. The planned 30%, 50%, and 80% isodose lines produced by the software for the water phantom (see start of Sec. IIB) were automically overlayed with the binned dose images of the gels.

**Figure 5:**
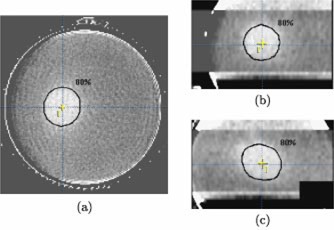
(Color) (a) Axial, (b) coronal, and (c) sagittal CT images of PAG2 (irradiated to a maximum dose of 8 Gy) at isocenter. The coronal and sagittal images were reconstructed from a set of axial gel images (3 mm slice spacing). The planned 80% isodose contour and a cross indicating the isocentre are marked in each image.

**Figure 6:**
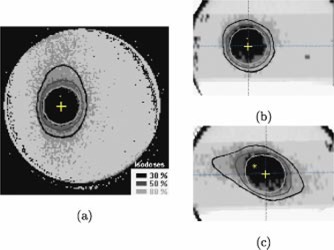
(Color) (a) Axial, (b) coronal, and (c) sagittal relative dose images of PAG1 at isocenter. These images were obtained by binning the grayscale images shown in Fig. 4 into four relative dose regions: <30%,30–50%,50–80%, and >80%. The planned 30%, 50%, and 80% isodose contours are shown in each image. The cross indicates the isocenter.

## III. RESULTS AND DISCUSSION

### A. High dose region localization

Figures 4 and 5 show an orthogonal set of planes [axial (a), reconstructed coronal (b), and reconstructed sagittal (c)] intersecting the measured relative dose volume, that has been co‐registered with the planned dose volume, for the PAG1 and PAG2 gels, respectively. All the planes intersect at the central axis, but all off‐axis planes may be scrolled through with the aid of movable dashed lines (see Fig. 4) featured in the software. The high dose volume created by the four arc irradiation is clearly visible as a hypo‐intense area in the relative dose images. The planned 80% isodose line is shown in black and is the peripheral isodose that we prescribe to at our clinic (determined once the maximum dose point is set to 100%). An initial qualitative comparison of the 80% isodose line with the hypo‐intense area indicates good agreement and ability of the CT gel technique to accurately localize the high dose regions produced by stereotactic irradiations.

In the axial views the 80% line is well centered within the hypo‐intense area of the PAG1 gel; however, the hypo‐intense area appears shifted by 1 mm in the PAG2 gel. The shift is comparable to a published average axial uncertainty of 1.3±1.3 mm for patients in the BrainLab® mask system.^24^ Greater shifting on the order of 3 mm is apparent in the coronal and sagittal views and may result in part from the effect of the 3 mm slice spacing on the resolution of the reconstructed views.

### B. Planned and measured dose comparison

The binned, measured dose information overlayed with the calcullated isodose lines is shown in Fig. 6 for PAG1. The planned and measured dose information agrees qualitatively in all planes. More precisely, the distance to agreement for the 80% and 50% isodose lines varies from 0–3 mm. Similar results were obtained for the same SRS planning/delivery system using film:^25^ 90% and 80% planned and measured isodose sufaces agreed within 1.5 mm and the 50% surfaces agreed within 2.5 mm.

The agreement between the planned and measured 30% isodose, however, is much poorer. The low dose gradient at 30% relative dose combined with the low signal‐to‐noise ratio causes scattering of the 30% relative dose value over a large area. In other words dose errors in low dose gradients translate into inherently poorer spatial definition of a particular relative dose value and distance to agreement measurements no longer pertain. Nonetheless, the shape and location of the planned and measured 30% relative dose information are qualitatively comparable.

For further comparison, the 80%, 50%, and 30% relative dose volumes of the PAG1 were calculated for both the planned and measured dose distributions. The planned dose volumes were obtained using a dose volume histogram for the whole volume of the gel and the planned volumes by summing the number of pixels with relative dose values greater than 30%, 50%, and 80%. The results are shown in Table I. The agreement is very good for the 80% and 50% relative doses, but less so for the 30% relative dose. The large discrepancy for the 30% dose volume is a reflection of the low dose gradient and low signal to noise ratio mentioned earlier.

**Table I:** Measured and calculated dose volumes for binned 15 Gy gel.

Isodose (%)	Measured volume (cm^3^)	Calculated volume (cm^3^)
80	14.0	14.6
50	30.5	27.1
30	83.2	53.4

For the PAG2, the lower dose contrast made the resolution of the binned relative dose ranges too poor to be useful. Thus, the more practical approach of not using a separate calibration gel to obtain relative dose information, but irradiating gels to doses within the linear portion of the response, is limited. Results may be improved by assuming a less conservative linear dose range (8 Gy) and irradiating gels to 11–12 Gy. This increase in maximum irradiation dose would increase the $\Delta N_{\text{CT}}$ signal by 30% and still permit extraction of relative dose information by simple ratios.

## IV. CONCLUSIONS

This paper reports on the application of a polymer gel x‐ray CT technique to 3D dose comparison with conformal radiotherapy planning delivery system. Two gels were irradiated with the same four arc stereotactic radiosurgery treatment: one gel to a maximum dose of 15 Gy (PAG1) and the second gel to a maximum dose of 8 Gy (PAG2). It was shown that CT imaging both gels with a noise reducing protocol yields good qualitative 3D dose information and allows for accurate localization of the high dose region. In the case of PAG1 accurate quantitative 3D dose information was also obtained, including 30%, 50%, and 80% isodose surfaces. A gel calibration curve was required for this gel, since the gel response is nonlinear at higher doses. PAG2, on the other hand, did not require a calibration curve since the gel response is linear to 8–10 Gy. However, the ease of dose information extraction for PAG2 comes at the expense of compromised dose information, due to the decrease in SNR in the gel. A practical advantage of the methods used in this work is that 3D evaluation of the measured dose volume was automatically accomplished by using the pre‐existing 3D SRS commercial treatment planning software. This method could be extended to non‐SRS treatment planning systems as well. Future work involves applications to more complex dose distributions, and improving dose resolution through the optimization of the gel composition to maximize signal obtained through x‐ray CT images with multiple detector scanners and higher capacity x‐ray tubes.^26^
